# Stretching of Mechanoreceptors in Superior Tarsal Muscle Reflexively Contracts Slow-Twitch Fibers in Facial Expression Muscles: A Case Series

**DOI:** 10.7759/cureus.64438

**Published:** 2024-07-12

**Authors:** Kiyoshi Matsuo, Ai Kaneko

**Affiliations:** 1 Plastic Surgery Oculoplastic Surgery, Matsuo Plastic and Oculoplastic Surgery Clinic, Hamamatsu, JPN; 2 Plastic and Reconstructive Surgery, Shinshu University School of Medicine, Matsumoto, JPN

**Keywords:** superior tarsal muscle, rostral locus coeruleus, mesencephalic trigeminal nucleus, trigeminal proprioception, blepharospasm, blepharoptosis, expressionless face, smiling, grimacing, facial expression muscle

## Abstract

Levator palpebrae superioris muscle (LPSM) and facial muscles comprise fast-twitch fibers (FTFs) and slow-twitch fibers (STFs) but lack muscle spindles required to contract STFs reflexively. Voluntary contractions and microsaccades of FTFs in LPSM stretch mechanoreceptors in superior tarsal muscle (STM) to induce phasic contractions of STFs in LPSM and frontalis muscle via mesencephalic trigeminal nucleus (MTN). They also induce prolonged contractions of STFs in bilateral frontalis and orbital orbicularis oculi muscles and physiological arousal via MTN and rostral locus coeruleus (LC). We hypothesized that stretching of mechanoreceptors in STM also induces prolonged contractions of STFs in other facial expression muscles (FEMs) via rostral LC. To verify this hypothesis, we reported a case series of abnormal contractions of FEMs due to aponeurosis disinsertion and disordered mechanoreceptor stretching. The first and second cases, which showed unilaterally and bilaterally sensitized mechanoreceptors, respectively, recorded increased prolonged contractions of ipsilateral and bilateral grimacing muscles, respectively. The third and fourth cases with asymmetrically and bilaterally desensitized mechanoreceptors experienced asymmetrically and bilaterally decreased prolonged contractions of grimacing and smiling muscles, respectively. Preoperatively and after surgery was performed to adjust mechanoreceptor stretching and reinsert aponeuroses into tarsi, we evaluated prolonged contractions of grimacing and smiling muscles during primary gazing and facial expression movements. Surgery satisfactorily cured abnormal prolonged contractions of grimacing and smiling muscles. Stretching of mechanoreceptors in STM by microsaccades or voluntary contractions of FTFs in LPSM might activate rostral LC via MTN, which tonically or phasically stimulates FEM motor neurons to reflexively contract their STFs, respectively.

## Introduction

Although the levator palpebrae superioris muscle (LPSM) and facial muscles comprise fast- and slow-twitch fibers and lack muscle spindles [1,2], which are required to induce involuntary reflex contraction of slow-twitch fibers [3], their fast- and slow-twitch fibers are contracted concurrently. Fast-twitch fibers in LPSM are derived embryologically from the global layer of the superior rectus muscle (GLSRM) [4]. The fast-twitch fibers in LPSM and GLSRM are both innervated by the same premotor neurons and contracted synchronously during vertical saccadic eye movements [5], whereas the slow-twitch fibers are skeletal, similar to those in the frontalis muscle that lacks muscle spindles. In this case series, we delineate the neural circuits that reflexively contract the slow-twitch fibers in the facial expression muscles (FEMs), which have not been well understood [2].

The superior tarsal muscle (STM) is innervated not only efferently by sparse, unmyelinated sympathetic nerve fibers but also afferently by abundant, myelinated trigeminal proprioceptive nerve fibers in a palisade arrangement, which function as mechanoreceptors [1,6]. These nerve fibers converge as a transverse nerve that runs in the space between the proximal STM and the distal belly of the LPSM, joining the medial branch of the lacrimal nerve in the first branch of the trigeminal nerve [1,6] to reach the mesencephalic trigeminal nucleus (MTN) [7]. Trigeminal proprioceptive neurons innervating mechanoreceptors in the STM at the MTN connect with rostral locus coeruleus neurons in a scattered manner through gap junctions [7]. 

Electrical stimulation of the unilateral transverse trigeminal proprioceptive nerve on the proximal STM induces phasic monosynaptic reflex contractions of slow-twitch fibers in the ipsilateral LPSM [8] and frontalis muscle via the MTN. It also induces prolonged polysynaptic reflex contractions of slow-twitch fibers in the bilateral frontalis muscles and orbital orbicularis oculi muscles (OOMs) with ipsilateral dominance via the MTN and rostral locus coeruleus for eye-eyelid-eyebrow coordinated movements [9,10].

Microsaccades are a type of fixational eye movement [11]. Involuntary, small contractions of the extraocular muscles at a frequency of 1-3 Hz displace the globe. This ensures that the vision does not fade during fixation because of neural adaptation and that trigeminal proprioception, activated by stretching the mechanoreceptors in the STM, does not fade while eyelids are kept open. Since skeletal muscle fibers in the LPSM interdigitated connect with smooth muscle fibers in the STM [12], upper eyelid opening with the microsaccades or the voluntary contractions of fast-twitch fibers in the LPSM with the GLSRM meticulously stretch the mechanoreceptors in the STM to tonically or phasically activate the rostral locus coeruleus via the MTN, respectively. The rostral locus coeruleus regulates physiological arousal, with prefrontal blood flow increase and sympathetic activation such as palmar sweating [13], which increases microsaccade velocity [11]. It also regulates prolonged reflex contractions of the slow-twitch fibers in the bilateral frontalis muscles and orbital OOMs with ipsilateral dominance [9,10]. 

We hypothesized that stretching of the mechanoreceptors in the STM might induce prolonged reflex contractions of slow-twitch fibers in other facial expression muscles (FEMs) in addition to the frontalis muscles and orbital OOMs via the MTN and rostral locus coeruleus [14].

To evaluate our hypothesis, we report a case series of four cases with abnormal contractions of FEMs due to aponeurosis disinsertion and disordered mechanoreceptor stretching. 

This article was presented at a symposium at the 33rd Research Council Meeting of the Japan Society of Plastic and Reconstructive Surgery on November 18-19th, 2021.

## Case presentation

**Figure 1:**
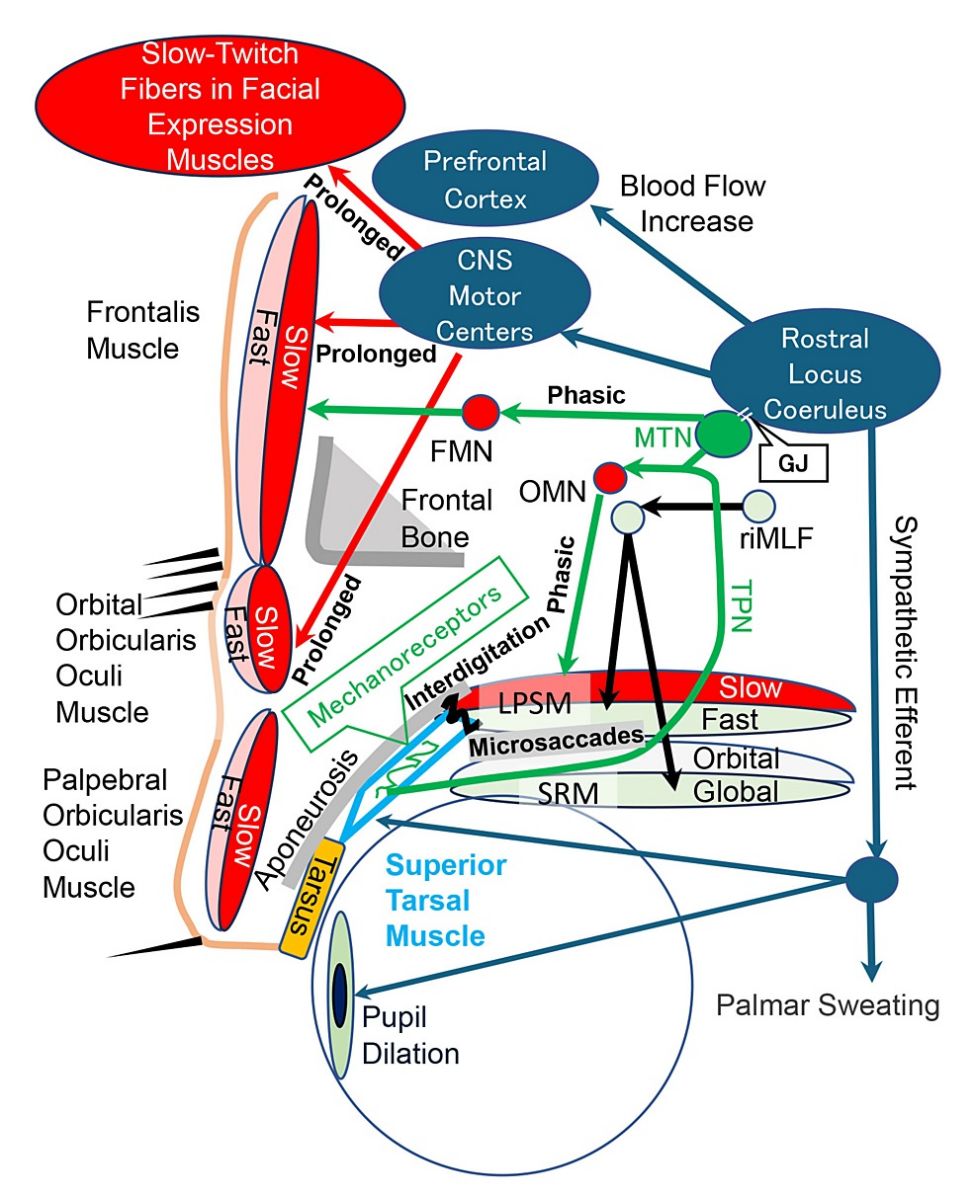
Diagram explaining the induced prolonged reflex contractions of slow-twitch fibers in facial expression muscles. The rostral interstitial nucleus of the medial longitudinal fasciculus (riMLF) and the oculomotor neurons (OMN) induce voluntary contractions and microsaccades of fast-twitch fibers (fast) in the levator palpebrae superioris muscle (LPSM) with the global layer of the superior rectus muscle (GLSRM). Skeletal muscle fibers of the LPSM interdigitated connect with smooth muscle fibers in the superior tarsal muscle (interdigitation). Thus, the voluntary contractions and the microsaccades of fast-twitch fibers in the LPSM meticulously stretch the mechanoreceptors in the superior tarsal muscle to induce phasic reflex contractions (phasic) of the slow-twitch fibers (slow) in the LPSM and frontalis muscle via the mesencephalic trigeminal nucleus (MTN). They also induce prolonged reflex contractions (prolonged) of slow-twitch fibers in the frontalis, orbital orbicularis oculi, and facial expression muscles, as well as physiological arousal with prefrontal blood flow increase and sympathetic activation, such as palmar sweating via the MTN and rostral locus coeruleus. The MTN connects with the rostral locus coeruleus through gap junctions (GJs). FMN: facial motor neuron, SRM: superior rectus muscle, TPN: trigeminal proprioceptive nerve. Image Credits: Kiyoshi Matsuo and Ai Kaneko.

Figure 1 presents the neuroanatomy of the prolonged reflex contractions of slow-twitch fibers in the FEMs as well as the frontalis muscle and orbital OOM, which are induced by stretching of the mechanoreceptors in the STM.

Methods

Preoperatively to establish whether the increased or decreased prolonged reflex contractions of slow-twitch fibers in the FEMs are caused by stretching of the sensitized or desensitized mechanoreceptors in the STM, we administered 4% lidocaine to anesthetize and desensitize the mechanoreceptors in the STM [15], or 1% phenylephrine to shorten the elongated STM and resensitize the mechanoreceptor in the STM [16], respectively. The patient was instructed to lie in a supine position, raise her/his chin, and gaze downward. The upper eyelid on the affected side was detached from the globe with a small retractor for 60 seconds to create a space in the upper fornix. Then, lidocaine or phenylephrine was administered into the space and was retained in this position by gravity to exclusively effect the mechanoreceptors in the STM.

The first and second cases with unilaterally and bilaterally sensitized mechanoreceptors experienced increased prolonged reflex contractions of the ipsilateral and bilateral grimacing muscles, respectively. The third and fourth cases with asymmetrically and bilaterally desensitized mechanoreceptors experienced asymmetrically and bilaterally decreased prolonged reflex contractions of the grimacing and smiling muscles, respectively. Preoperatively and six months after surgery that was performed to adjust the mechanoreceptor stretching and reinsert the aponeuroses into the tarsi, we evaluated the prolonged reflex contractions of the slow-twitch fibers in the FEMs due to ocular, palpebral, and facial movements. Case 1 was asked to maintain the primary gaze, maximum upgaze, and maximum tight eyelid closure unilaterally and bilaterally. Case 2 was asked to maintain the primary gaze and maximum upgaze. Cases 3 and 4 were asked to maintain the primary gaze, maximum tight eyelid closure or frown, and maximum smile.

This study was approved on February 2, 2016, by the Shinshu University School of Medicine Biological and Medical Research Ethics Committee (Permission number: 30869). Verbal or written informed consent was obtained from the patients for the publication of this case report and accompanying images.

Case 1 

**Figure 2:**
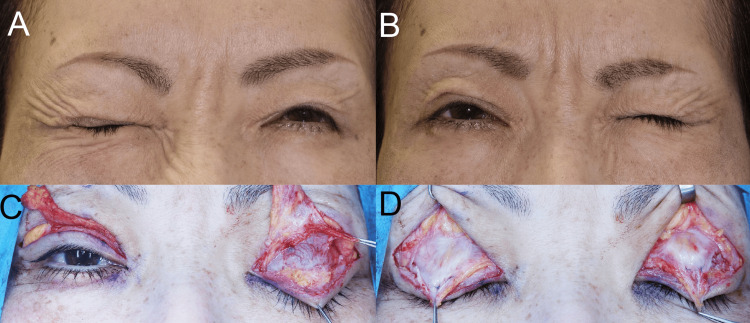
Case 1. (A) and (B) Unilateral tight eyelid closure indicated that the left facial motor neurons of the muscles for eyelid closure were more innervated by the bilateral motor cortices than by the right ones. (C) The left hypertrophic superior tarsal muscle, with lamina propria mucosae, was dissected from the aponeurosis, tarsus, and conjunctiva palpebrae and was pinched by forceps. (D) After releasing the lateral horns, the aponeuroses without the superior tarsal muscle were fixed to the tarsi.

A 49-year-old woman presented with unilateral blepharospasm worsened by upgaze (Video 1). Unilateral tight eyelid closure and subsequent changes in contralateral contractions of the orbital OOM and corrugator supercilii muscle revealed that the left facial motor neurons of these muscles were more innervated by the bilateral motor cortices than by the right ones [17] (Figures 2A, 2B). Thus, the activities of the left orbital OOM and corrugator supercilii muscle increased and ipsilaterally antagonized the opening of the left upper eyelid. Asymmetrically enhanced mechanoreceptor stretching due to the asymmetrically hyperactive orbital OOMs induced asymmetrically increased reflex contractions of the orbital OOM and corrugator supercilii muscle, similar to unilateral blepharospasm. Surgery was performed to unilaterally desensitize mechanoreceptors in the STM [15] (Figure 2C) and fix the aponeuroses to the tarsi [16] (Figure 2D). This reduced the prolonged reflex contractions of the ipsilateral grimacing muscles, curing the unilateral blepharospasm, which was not worsened by upgaze (Video 2).

**Video 1:** Case 1. Preoperative condition. Left blepharospasm was notably worsened by upgazing but was less worsened by tight eyelid closure.

**Video 2:** Case 1. Postoperative condition. The left blepharospasm was not induced by upgazing or tight eyelid closure.

Case 2

**Figure 3:**
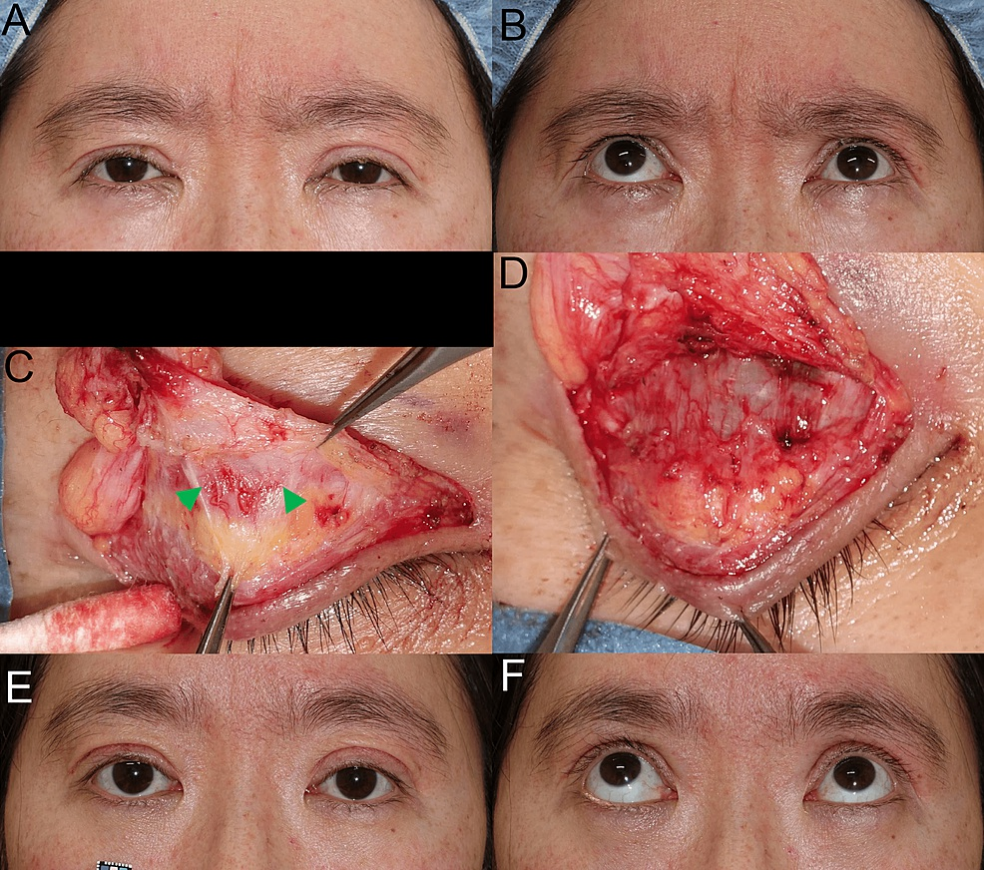
Case 2. (A) and (B) Preoperatively, grimacing (bilateral blepharospasm) during primary gazing was worsened by upgazing. (C) and (D) Because the buried sutures were entangled with abundant, myelinated proprioceptive nerve fibers and sensitized mechanoreceptors in STM, STM with scar tissue (green arrowheads) and lamina propria mucosae was dissected from the aponeurosis, tarsus, and conjunctiva palpebrae to facilitate mechanoreceptor stretching. After releasing the lateral horns, the aponeuroses without the STM were fixed to the tarsi. (E) and (F) Postoperatively, the grimacing was cured and was not worsened by upgazing. STM: superior tarsal muscle.

A 44-year-old woman presented with involuntary grimacing during primary gazing, which was worsened by upgazing (Figures 3A, 3B). This grimacing was caused by the surgery for creating a superior palpebral crease and fixing the levator aponeurosis to raise the upper eyelid, in which the sutures were buried between the upper conjunctiva palpebrae and the pretarsal skin through the STM and the aponeurosis. We diagnosed that symmetrically enhanced mechanoreceptor stretching due to the bilaterally sutured STMs induced symmetrically increased prolonged reflex contractions of the grimacing muscles, similar to bilateral blepharospasm. Surgery was performed to bilaterally desensitize mechanoreceptors in STM as follows [15]: we released the scar tissue in the STMs to facilitate stretching of the mechanoreceptors in STM (Figures 3C, 3D). After releasing the lateral horns, the aponeuroses were fixed to the anterior tarsi [16]. Surgery cured the increased prolonged reflex contractions of the bilateral grimacing muscles (Figures 3E, 3F). 

Case 3

**Figure 4:**
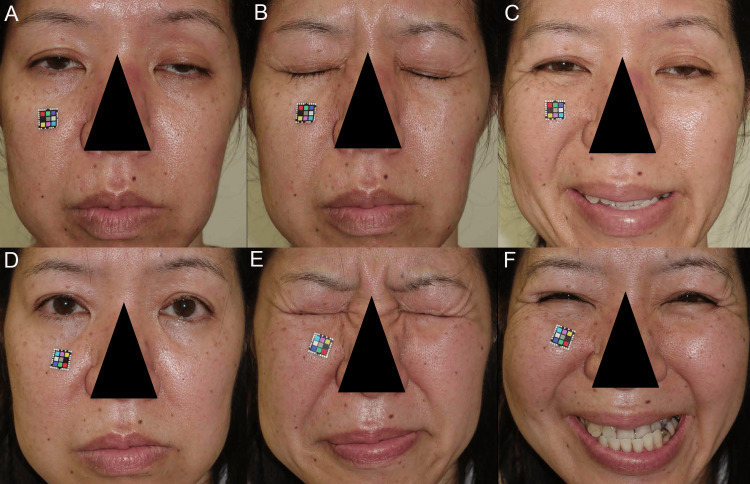
Case 3. (A) Preoperatively, primary gazing revealed asymmetrical blepharoptosis and an expressionless face. (B) Preoperatively, she could not perform grimacing and frowning. We asked her to close the bilateral eyelids tightly, but she could not. (C) Preoperatively, her chief complaint was a smile-less expression on the left face. (D) Postoperatively, primary gazing showed an expressive face, and her facial expression suggested that all slow-twitch fibers in the FEMs were restored. (E) Postoperatively, she could fully perform bilateral tight eyelid closure with grimacing. (F) She was also able to grin symmetrically. FEMs: facial expression muscles.

A 45-year-old woman presented with asymmetrically worsened blepharoptosis and an expressionless face (specifically smile-less) face without deepening of the nasolabial fold (Figures 4A-4C). She was considered not to have left facial palsy but was diagnosed with impaired prolonged reflex contractions of the slow-twitch fibers in the FEMs because of advanced asymmetrical blepharoptosis. Asymmetrically reduced mechanoreceptor stretching due to asymmetrical aponeuroses disinsertion from the tarsi and elongation of the STMs caused asymmetrical blepharoptosis [16] and the expressionless face. Surgery performed to symmetrically fix the disinserted aponeuroses to the tarsi restored symmetrical prolonged reflex contractions of slow-twitch fibers in the LPSM [16] and FEMs for eyelid opening, grimacing, and smiling (Figures 4D-4F). She was satisfied, especially regarding the possible deepening of the left nasolabial fold to enable smiling.

Case 4

**Figure 5:**
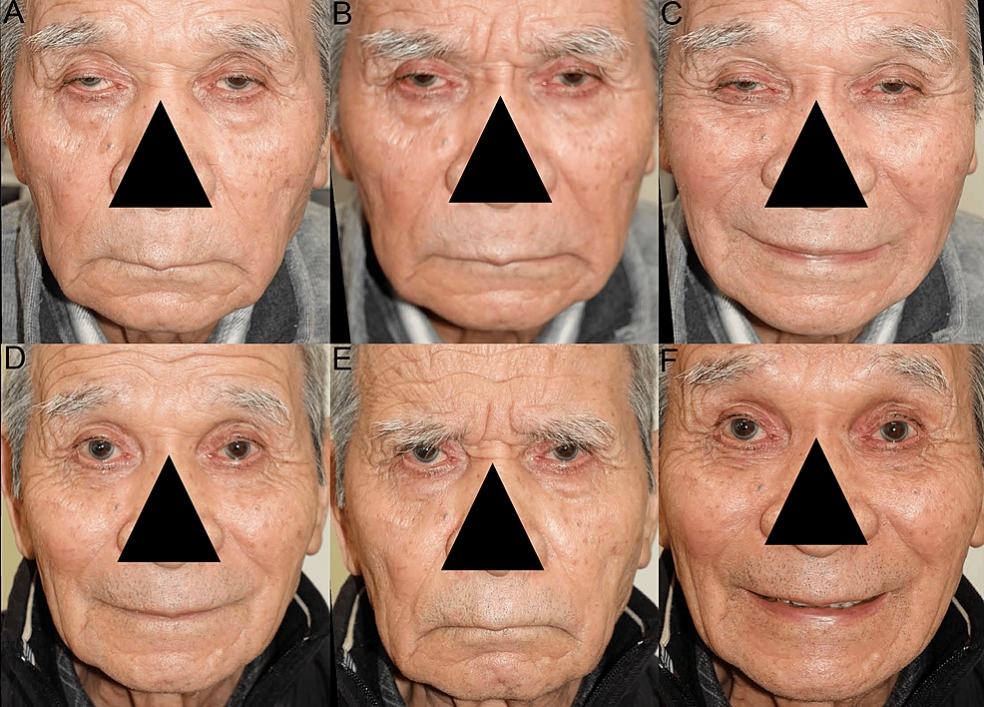
Case 4. (A) Preoperatively, primary gazing revealed bilateral blepharoptosis and an expressionless face. (B) and (C) Preoperatively, the patient could slightly frown and smile, probably because he could voluntarily contract the fast-twitch fibers in the frowning and smiling muscles. (D) Postoperatively, primary gazing showed an expressive face, and the appearance of his face indicated that prolonged reflex contractions of most slow-twitch fibers in the FEMs, including muscle fibers in the superficial musculoaponeurotic system, were significantly restored. (E) and (F) Postoperatively, his frowning and smiling suggested that fast-twitch fibers and slow-twitch fibers in the FEMs were contracted concurrently. FEMs: facial expression muscles.

An 85-year-old man presented with bilaterally worsening blepharoptosis and an expressionless sagging face (Figures 5A-5C). We diagnosed that his facial sagging was caused by elongation of the facial retaining ligaments and severe blepharoptosis. Symmetrically reduced mechanoreceptor stretching due to symmetrical aponeuroses disinsertion from the tarsi and elongation of STMs caused the bilateral blepharoptosis [16] and the expressionless sagging face. Surgery performed to bilaterally fix the disinserted aponeuroses to the tarsi restored bilateral reflex contractions of slow-twitch fibers in LPSM [16] and FEMs for eyelid opening, frowning, and maximum smiling with visible teeth (Figures 5D-5F). He and his family were satisfied with the restored tones of the FEMs, including the muscle fibers in the superficial muscular aponeurotic system, which supports the sagging facial skin.

## Discussion

During facial expressions, such as grimacing, tight eyelid closure, frowning, and maximum smiling, both the fast-twitch fibers and the slow-twitch fibers in the FEMs are contracted concurrently. Phasic voluntary contractions of the fast-twitch fibers in FEMs are induced by cortico-facial projections. However, prolonged reflex contractions of the slow-twitch fibers in FEMs appeared to be induced by the stretching of the mechanoreceptors in the STM due to phasic voluntary contractions and/or microsaccades of fast-twitch fibers in the LPSM with the GLSRM via the MTN and rostral locus coeruleus (Figure 1).

Preoperatively, in cases 1 and 2, during primary gazing (Video 1 and Figure 3A), because the microsaccades of fast-twitch fibers in the LPSM with GLSRM stretched the unilaterally and bilaterally sensitized mechanoreceptors in the STM, the prolonged reflex contractions of the slow-twitch fibers in the ipsilateral and bilateral frontalis muscles and orbital OOMs were increased via the MTN and rostral locus coeruleus (Figure 1) [9,10]. This resulted in unilateral and bilateral grimacing, respectively.

During maximum upgazing (Video 1 and Figure 3B), the increased phasic voluntary contractions and microsaccades of fast-twitch fibers in the LPSM with GLSRM further stretched the unilaterally and bilaterally sensitized mechanoreceptors in the STM. Thus, the prolonged reflex contractions of slow-twitch fibers in the ipsilateral and bilateral frontalis muscles and orbital OOMs were further increased via the MTN and rostral locus coeruleus (Figure 1), aggravating the unilateral and bilateral grimacing.

In case 1, bilateral tight eyelid closure (Video 1) induced phasic voluntary contractions of fast-twitch fibers in the orbital OOMs and reflex upward movement of the globe (Bell's phenomenon). Because the reflex upward movement of the globe increased the phasic reflex contractions and microsaccades of fast-twitch fibers in the LPSM with GLSRM, the prolonged reflex contractions of slow-twitch fibers in the bilateral orbital OOMs were increased via the MTN and rostral locus coeruleus in the same manner as upgazing. However, because the extent of mechanoreceptor stretching during tight eyelid closure was less than that during maximum upgazing, the prolonged reflex contractions of slow-twitch fibers in the orbital OOMs were less during tight eyelid closure than during upgazing (Video 1).

In case 1, unilateral tight eyelid closure with the opening of the contralateral eyelid (Figures 2A, 2B) appeared to induce not only the phasic voluntary contractions of the fast-twitch fibers in the ipsilateral orbital OOM but also the reflex upward movement of the globe (Bell's phenomenon) with the antagonizing downward movement of the globe by phasic reflex contractions of fast-twitch fibers in the inferior rectus muscle (GLIRM) to maintain the visual axis of the bilateral eyes. This led to the bilateral globes being pulled backward. Moreover, the increased phasic reflex contractions and microsaccades of the fast-twitch fibers in the LPSM with GLSRM in the ipsilateral eyelid might increase stretching of the mechanoreceptors in STM to increase the prolonged reflex contractions of slow-twitch fibers in the orbital OOM via the MTN and rostral locus coeruleus, resulting in tight eyelid closure.

In cases 1 and 2 after surgery, during primary gazing (Video 2 and Figure 3E), the microsaccades of fast-twitch fibers in the LPSM with GLSRM stretched the unilaterally or bilaterally desensitized mechanoreceptors in the STM. As a result, the prolonged reflex contractions of slow-twitch fibers in the ipsilateral and bilateral orbital OOMs were decreased via the MTN and rostral locus coeruleus, resulting in the unilateral and bilateral absence of grimacing, respectively.

During maximum upgazing (Video 2 and Figure 3F), phasic voluntary contractions and microsaccades of fast-twitch fibers in the LPSM with GLSRM were not increased due to the reduction in the functional resistance of the upper eyelid during primary gazing, and the mechanoreceptors in the STMs were unilaterally and bilaterally desensitized. Consequently, the prolonged reflex contractions of slow-twitch fibers in the ipsilateral and bilateral orbital OOMs by mechanoreceptor stretching were decreased via the MTN and rostral locus coeruleus, curing the unilateral and bilateral grimacing, respectively.

Preoperatively, in cases 3 and 4, during primary gazing (Figures 4A, 5A), microsaccades of the fast-twitch fibers in LPSM with GLSRM did not effectively stretch the asymmetrically and bilaterally desensitized mechanoreceptors in the STM. This resulted in the asymmetrical and bilateral decrease in prolonged reflex contractions of the slow-twitch fibers in FEMs via the MTN and rostral locus coeruleus, resulting in the asymmetrical and bilateral expressionless faces, respectively.

In case 3, during bilateral tight eyelid closure (Figure 4B), the reflex upward movement of the globes (Bell's phenomenon) increased the phasic reflex contractions and microsaccades of fast-twitch fibers in LPSM with GLSRM, which did not effectively stretch the asymmetrically desensitized mechanoreceptors in the STMs. As such, the prolonged reflex contractions of the slow-twitch fibers in FEMs were not increased via the MTN and rostral locus coeruleus, disabling tight eyelid closure.

In case 4, during frowning with the opening of eyelids (Figure 5B), the backward pull of the globes probably increased the phasic reflex contractions and microsaccades of the fast-twitch fibers in both the LPSM with GLSRM and the global layer of the inferior rectus muscle (GLIRM), which did not effectively stretch the bilaterally desensitized mechanoreceptors in the STM. As a result, the prolonged reflex contractions of slow-twitch fibers in the frowning muscles were not satisfactorily increased via the MTN and rostral locus coeruleus.

During maximum smiling in cases 3 and 4 (Figures 4C, 5C), the backward pull of the globes possibly increased the phasic reflex contractions and microsaccades of fast-twitch fibers in the LPSM with GLSRM and the GLIRM, which did not effectively stretch the asymmetrically and bilaterally desensitized mechanoreceptors in the STM. This may have led to the non-satisfactory increase in the prolonged reflex contractions of slow-twitch fibers in the smiling muscles via the MTN and rostral locus coeruleus, respectively.

Postoperatively, in cases 3 and 4, during primary gazing (Figures 4D, 5D), the microsaccades of fast-twitch fibers in the LPSM with GLSRM stretched the bilaterally resensitized mechanoreceptors in the STMs. This led to the restoration of the prolonged reflex contractions of slow-twitch fibers in the FEMs via the MTN and rostral locus coeruleus, resulting in the expressive faces.

In case 3, during bilateral tight eyelid closure (Figure 4E), the reflex upward movement of the globes (Bell's phenomenon) increased the phasic reflex contractions and microsaccades of fast-twitch fibers in the LPSM with GLSRM and the GLIRM, which effectively stretched the bilaterally resensitized mechanoreceptors in the STMs. Consequently, the prolonged reflex contractions of slow-twitch fibers in the FEMs were increased symmetrically via the MTN and rostral locus coeruleus, contracting all the FEMs.

In case 4, during frowning (Figure 5E), the backward pull of the globes probably increased the phasic reflex contractions and microsaccades of fast-twitch fibers in the LPSM with GLSRM and the GLIRM, which stretched the bilaterally resensitized mechanoreceptors in the STMs. As such, the prolonged reflex contractions of slow-twitch fibers in the frowning muscles (the corrugator supercilii muscles and orbital OOMs) were increased via the MTN and rostral locus coeruleus.

During maximum smiling (Figures 4F, 5F), the backward pull of the globes probably increased the phasic reflex contractions and microsaccades of fast-twitch fibers in the LPSM with GLSRM and the GLIRM, which stretched the bilaterally resensitized mechanoreceptors in the STMs. This led to an increase in the prolonged reflex contractions of slow-twitch fibers in the bilateral smiling muscles via the MTN and rostral locus coeruleus, resulting in joyful smiles.

The omega and Veraguth triangular folds as the grimacing in the glabella, as shown in case 2 (Figure 3A), are a physical sign of depression with lower prefrontal blood flow [18]. The increased phasic voluntary contractions and microsaccades of the fast-twitch fibers in the LPSM with GLSRM stretch the mechanoreceptors in STMs not only to activate the prefrontal cortex to increase the cerebral blood flow but also to increase the prolonged reflex contractions of slow-twitch fibers in the frontalis muscles and orbital OOMs via the MTN and rostral locus coeruleus (Figure 1), resulting in the glabellar grimacing. The turned-down corners of the mouth due to the expressionless face, as shown in case 4 (Figure 5A) are another physical sign of depression [18]. The prolonged reflex contractions of STFs in the FEMs for raising-up corners of the mouth are not increased by reduced stretching of the mechanoreceptors in the STMs via the MTN and rostral locus coeruleus, which did not activate the prefrontal cortex to increase the cerebral blood flow. Facial grimacing for pain modulation is used in the Glasgow coma scale as an arousal reaction and in pain scales for various animals and is induced by enhanced stretching of the mechanoreceptors in STMs via the MTN and rostral locus coeruleus.

The orbital OOM has more slow-twitch fibers than the inner palpebral OOM, whereas the innermost fascicles of the orbicularis oris muscle are entirely formed by slow-twitch fibers in contrast with the remaining muscle where slow-twitch fibers represent only 30% of the total, indicating a functional subdivision within the muscles [2]. Thus, the frowning in case 4 (Figures 4B, 4E) appeared to consist of both increased prolonged reflex contractions of the slow-twitch fibers in the orbital OOM for squeezing the palpebral fissure and increased prolonged reflex contractions of the slow-twitch fibers in the inner orbicularis oris muscle for pursing the lip. Therefore, the rostral locus coeruleus projection to the CNS facial motor control center to contract the orbital OOM and that to contract the inner orbicularis oris muscle might be functionally connected for frowning.

The maximum smiling (grinning) in case 3 (Figure 4F) appeared to consist of various reflex contractions of slow-twitch fibers in the palpebral OOM for narrowing the palpebral fissure without contractions of the corrugator supercilii muscle and orbital OOM, the levator anguli oris muscle for raising the mouth corner, the depressor labii inferioris muscle for pulling down the lower lip, and the zygomatic major, zygomatic minor, levator labii superioris, and levator labii superioris alaeque nasi muscles for deepening the nasolabial fold. The rostral locus coeruleus projections to the CNS motor centers for smiling to simultaneously contract each of the many smiling muscles might also be functionally connected [14]. The tight eyelid closure in case 3 (Figure 4E) appeared to consist of prolonged reflex contractions of slow-twitch fibers in the frowning and smiling muscle groups.

The facial sagging in case 4 (Figure 5A) appeared to be caused by the facial droop due to decreased reflex contractions of slow-twitch fibers in the FEMs and by the facial skin sagging due to elongation of the facial retaining ligaments and superficial musculoaponeurotic system (SMAS). Surgery restored the facial droop, produced an expressive face, and cured the facial skin sagging (Figure 5D). As a result, the restored stretching of mechanoreceptors in the STM may contract the muscle component in SMAS, raising the facial skin.

Although the proportions of fiber types in the facial muscles have been reported [19], all slow-twitch fibers in the facial muscles are not reflexively contracted by stretching of mechanoreceptors in the STM. For instance, slow-twitch fibers in the LPSM, as the major eyelid opening muscle, are reflexively contracted by stretching of mechanoreceptors in the STM. On the other hand, slow-twitch fibers in the palpebral OOM, as the major eyelid closing muscle, are reflexively contracted by stretching of putative mechanoreceptors in the inferior tarsal muscle (Figure 1) [20].

The locus coeruleus sends projections to the forebrain, brainstem, cerebellum, and spinal cord to regulate arousal, sympathetic tone, posture muscle tone, and pain modulation [13,14]. The four cases indicated that the rostral locus coeruleus, activated by stretching of mechanoreceptors in the STM due to phasic voluntary contractions and microsaccades of the fast-twitch fibers in the LPSM with GLSRM, appeared to send projections to the CNS motor centers involving the facial motor nucleus to contract the slow-twitch fibers in the FEMs for grimacing, tight eyelid closure, frowning, and smiling muscles in addition to the frontalis muscle and orbital OOM as the eye-eyelid-eyebrow-face coordinated movements. For instance, because maximum upgaze tilts the head and deforms the body with bending of the knee joint, stretching the mechanoreceptors in the STM might reflexively contract muscles involved in head and body movements as eye-eyelid-eyebrow-face-head-body coordinated movements. Rostral locus coeruleus projections to CNS motor centers should be investigated.

Facial expressions are supposed to reflect a person’s mood. Because upgaze, which induces grimacing in the glabella, increases physiological arousal with prefrontal blood flow increase and sympathetic activation such as palmar sweating, it should be assessed how facial expressions, such as grimacing, tight eyelid closure, frowning, and smiling, affect prefrontal blood flow and sympathetic activation.

## Conclusions

Eyelid opening with microsaccades or phasic voluntary contractions of the fast-twitch fibers in LPSM with GLSRM meticulously stretches the mechanoreceptors in STM for trigeminal proprioceptive activation. Thus, this proprioception might tonically or phasically activate the rostral locus coeruleus, which regulates the prolonged reflex contractions of slow-twitch fibers in facial expression muscles for grimacing, tight eyelid closure, frowning, and smiling, as well as the physiological arousal.
